# Increasing the Use of Newborn Pain Treatment Following the Implementation of a Parent-Targeted Video: An Outcome Evaluation

**DOI:** 10.3390/children11111360

**Published:** 2024-11-09

**Authors:** Michaela A. Smith, Sandra I. Dunn, Catherine Larocque, Jodi Wilding, Marsha Campbell-Yeo, Lucy Gilmore, JoAnn Harrold, Jiale Hu, Carolina Lavin Venegas, Shokoufeh Modanloo, Stuart G. Nicholls, Pat O’Flaherty, Shahirose Sadrudin Premji, Jessica Reszel, Sonia Semenic, Janet E. Squires, Bonnie Stevens, Marie-Josee Trepanier, Kathy Venter, Denise Harrison

**Affiliations:** 1 Better Outcomes Registry & Network (BORN) Ontario, Ottawa, ON K1H 8L1, Canada; sdunn@bornontario.ca (S.I.D.); clavinvenegas@bornontario.ca (C.L.V.);; 2 Children’s Hospital of Eastern Ontario (CHEO) Research Institute, Ottawa, ON K1H 8L1, Canada; 3 School of Nursing, University of Ottawa, Ottawa, ON K1N 6N5, Canada; claro007@uottawa.ca (C.L.);; 4 School of Nursing, Dalhousie University, Halifax, NS B3H 4R2, Canada; marsha.campbell-yeo@dal.ca; 5 MOM-LINC Lab, IWK Health Centre, Halifax, NS B3K 6R8, Canada; 6 Headwaters Health Care Centre, Orangeville, ON L9W 4X9, Canada; lucygilmore13@gmail.com; 7 Department of Pediatrics, Children’s Hospital of Eastern Ontario (CHEO), Ottawa, ON K1H 8L1, Canada; jharrold@cheo.on.ca; 8 Department of Obstetrics, Gynecology and Newborn Care, The Ottawa Hospital, Ottawa, ON K1H 8L6, Canada; 9 Department of Nurse Anesthesia, Virginia Commonwealth University, Richmond, VA Box 980226, USA; jhu4@vcu.edu; 10 Arthur Labatt Family School of Nursing, Western University, London, ON N6A 3K7, Canada; smoda044@uottawa.ca; 11 Ottawa Hospital Research Institute (OHRI), Ottawa, ON K1H 8L6, Canada; snicholls@ohri.ca; 12 Champlain Maternal Newborn Regional Program, Ottawa, ON K1G 4J8, Canadamjtrepanier@rogers.com (M.-J.T.); 13 School of Nursing, Faculty of Health Sciences, Queen’s University, Kingston, ON K7L 3N6, Canada; shahirose.premji@queensu.ca; 14 Ingram School of Nursing, McGill University, Montréal, QC H3A 2M7, Canada; sonia.semenic@mcgill.ca; 15 Research Institute, The Hospital for Sick Children, Toronto, ON M5G 0A4, Canada; 16 Lawrence Bloomberg Faculty of Nursing, The University of Toronto, Toronto, ON M5T 1P8, Canada; 17 Breastfeeding Committee for Canada (BCC), Baby Friendly Initiative (BFI) Assessment Committee, Glen Margaret, Halifax, NS B3Z 3H8, Canada; 18 Department of Nursing, University of Melbourne, Parkville, VIC 3053, Australia; 19 Murdoch Children’s Research Institute, Melbourne, VIC 3052, Australia; 20 Royal Children’s Hospital, Melbourne, VIC 3052, Australia

**Keywords:** infant, newborn, pain, breastfeeding, family, education

## Abstract

Background/Objectives: Despite strong evidence that breastfeeding, skin-to-skin care, and sucrose reduce pain in newborns during minor painful procedures, these interventions remain underutilized in practice. To address this knowledge-to-practice gap, we produced a five-minute parent-targeted video demonstrating the analgesic effects of these strategies and examined whether the use of newborn pain treatment increased in maternal–newborn care settings following the introduction of the video by nurses. Methods: The design was a pre–post outcome evaluation. The participants were infants born in eight maternal–newborn hospital units in Ontario, Canada. Data on newborn pain treatment were obtained from a provincial birth registry. Descriptive statistics and chi square tests were used to compare the before-and-after changes in the use of pain treatment. Results: Data on 15,524 infants were included. Overall, there was an increase in the proportion of newborns receiving any pain treatment comparing before (49%) and after (54%) the video intervention (*p* < 0.0001) and a decrease in the proportion of newborns receiving no pain treatment pre- (17.6%) and post-intervention (11.5%) (*p* < 0.0001). Most of the change aligned with increased sucrose use (35% to 47%, *p* < 0.0001) in three of the larger units. Nevertheless, considerable increases in the use of breastfeeding and/or skin-to-skin care (24% to 38%, *p* < 0.0001) were also observed in three of the smaller units. Conclusions: The video intervention was effective at increasing the use of pain treatment for newborns. Though the overall increases were modest, there were some large increases for specific methods of pain treatment in certain maternal–newborn units, reflecting the diversity in practice and context across different sites.

## 1. Introduction

Newborn infants undergo routine capillary or venous blood sampling in the first days of life for newborn screening, and preterm infants and those with illnesses often require multiple blood samplings and other painful procedures during hospitalization [1,2]. Given the frequent need for blood sampling, effective pain management is crucial. High-quality synthesized evidence exists for analgesic effects of breastfeeding, when feasible and culturally acceptable [3], skin-to-skin care [4] and small amounts of sweet solutions, either sucrose or glucose [5,6], during minor painful procedures. These strategies are also now recommended in the recently published Canadian national pediatric pain standard for acute procedural pain in infants [7]. Nevertheless, these approaches are inconsistently used in practice in neonatal intensive care units (NICUs) [1,2], and for healthy term infants in maternal–newborn birthing units [8].

A key agenda in neonatal procedural pain management is to increase the uptake of evidence-based practice [9]. In an effort to improve the implementation of evidence-based strategies for neonatal pain management, the study team, in partnership with parents and clinicians, co-produced a brief parent-targeted educational video, titled Be Sweet to Babies (BSweet2Babies: https://youtu.be/L43y0H6XEH4, accessed on 1 October 2024) describing three different newborn pain management strategies [10]. The English version of this video is 4 min, 23 s, and shows two babies having a heel prick while being breastfed or held in skin-to-skin care, as well as a third newborn undergoing venipuncture and receiving sucrose. The voice-over in user-friendly language explains how parents can work with clinicians to use these strategies during minor painful procedures to reduce their baby’s pain [11].

The rationale for creating a video intervention was that written information is known to have limited effectiveness in changing practices and over many years, healthcare provider education and implementation attempts to improve parental involvement have been of limited success [10]. In addition, very little attention had been directed towards parents in the translation of knowledge of best pain management practices, yet parents are needed to breastfeed and hold skin-to-skin. Therefore, the aim of the video was to visually demonstrate the effectiveness of parent involvement—breastfeeding and skin-to-skin care, as well as sucrose—during actual painful procedures. Previous evaluations have focused on the video’s reach, viewers’ knowledge, as well as previous use and future intent to apply the three strategies [11]. However, the actual adoption of these strategies into widespread clinical practice has not been ascertained. Furthermore, it was unclear whether providing maternal–newborn care hospitals with the parent-targeted BSweet2Babies video would result in a measurable increase in the use of these evidence-based pain treatment approaches.

The aim of this study was therefore to evaluate whether there was any change in the proportion of infants who received pain relief (breastfeeding, skin-to-skin care, and/or sucrose) during newborn blood sampling in the period beginning 6 months after the implementation of the video in maternal–newborn care settings.

## 2. Materials and Methods

### 2.1. Overview of Study and Data Source

This study is reported in accordance with the REporting of studies Conducted using Observational Routinely collected Data (RECORD) guidelines [12]. This analysis is the quantitative portion of a larger exploratory mixed-methods study, which included a qualitative evaluation [13], followed by an analysis of survey data collected from nursing staff leaders and clinical staff regarding implementation of the video [14]. This paper reports on the final outcomes of the study, where we assessed implementation effectiveness in terms of whether providing hospital maternal–newborn units with the BSweet2Babies video led to an increase in the use of pain treatment (breastfeeding, skin-to-skin care, or sucrose) during newborn blood sampling.

Data for this evaluation came from Better Outcomes Registry & Network (BORN) Ontario, a provincial birth registry that systematically collects information on virtually every birth in Ontario (population ~15 million) for the purposes of facilitating and improving maternal–newborn care [15]. Data are collected in near real-time either by point-of-care manual data entry into a secure portal, direct feeds from hospital systems, or by automated extraction and batch uploads from electronic health record systems. A robust linking and matching algorithm ensures data sources are appropriately aggregated to individual records. The routine data collected include clinical information on the pregnancy, the birth, and the newborn, and data quality assessments have concluded that these data are highly reliable [16,17,18]. All data collected by BORN Ontario are held in a secure cloud-based database called the BORN Information System (BIS), from which records can be extracted for analysis.

### 2.2. Study Recruitment and Description of Intervention

Methods for the full study are reported in the trial registry (ClinicalTrials.gov NCT03099252, 1 September 2018) and in previous reports [13,14]. To briefly summarize, inpatient hospital units providing maternal–newborn care across the province of Ontario, Canada, were targeted for recruitment. Hospitals were eligible for inclusion if they (i) provided Level 1 or Level 2 maternal and newborn care [19]; (ii) had a birth volume of at least 50 infants per year; (iii) had ≤85% use of pain management (breastfeeding, skin-to-skin care, sucrose) during newborn screening or bilirubin sampling, as per baseline pain management data in the BIS [20]; and (iv) had ≤50% missing data for the newborn pain management data element in the BIS. All eligible units were invited to participate. The study processes were pilot-tested at two hospitals, which were then considered ineligible for participation in the trial. A total of 47 units were eligible for inclusion, and eight maternal–newborn units were ultimately enrolled into the study, after 19 units declined to participate and 20 units had no response or were lost to follow-up during recruitment [13]. Two participating sites were part of the same hospital corporation but, otherwise, the sites were geographically distributed throughout Ontario, representing a mix of urban and rural hospitals as well as locations in the north, south, east, and west of the province.

As reported previously [13], the designated nurse unit leader at all enrolled sites received the following tools and resources as implementation strategies to facilitate use of the video intervention:

1. An electronic tablet preloaded with the parent-targeted BSweet2Babies video (in 10 different languages: English, French, Arabic, German, Hindi, Inuktitut, Mandarin, Farsi, Portuguese, Spanish);
2. Parent cards—visual reminder for parents with a QR code that directs them to the video on YouTube;
3. BSweet2Babies Poster—visual reminder for parents and healthcare providers on the enrolled units, which included the same QR code and URL;
4. Monthly support calls for the nursing leaders/delegate of the maternal–newborn units;
5. Bi-monthly community of practice teleconferences for the nursing leaders of the units to discuss barriers and facilitators to video implementation and use of newborn pain management.

These resources were included as supplemental files at each site [13]. Participating units were asked to offer the video to all parents during the 6-month intervention period and beyond (using the preferred delivery methods of the hospital and parents). The video was available via multiple means to facilitate optimal parental exposure across settings.

### 2.3. Statistical Analysis and Outcomes

**Figure 1:**
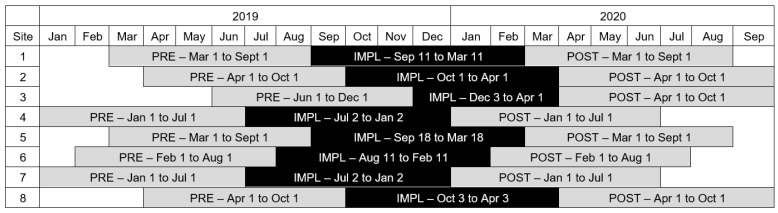
Timeline of pre-intervention period (PRE), implementation period (IMPL), and post-implementation period (POST) for each participating site.

Data for this evaluation were extracted from the BIS on 1 April 2021. To create the analytical cohort, we included live born infants at each of the participating maternal–newborn units in the 6-month period prior to implementation of the video intervention as well as the 6-month period after implementation. Further inclusion criteria were then applied to the analytical cohort, restricted to only those infants who underwent newborn screening or serum bilirubin testing in hospitals. Infant records were also excluded if the birth hospital differed from the hospital submitting data to the BIS, as this could mean an infant was born at a hospital that was not participating in the study. The implementation periods were slightly different for each unit due to ethics approval processes, but all the pre-intervention periods were in 2019 and all the post-intervention periods were in 2020 (Figure 1). Of note, site 3 had a slightly shorter implementation period than other sites (only 4 months) as they started later in December 2019 and then had to put all non-essential research on hold once the COVID-19 pandemic started in March 2020. 

The primary outcome for this evaluation was the proportion of infants who received any of the three evidence-based pain treatment methods before and after the implementation of the video intervention. To ascertain this, we used information from the ‘Pain relief measures during first blood sampling by heel prick’ data element in the BIS, which captures the type(s) of pain relief used for each infant during newborn blood sampling in either the birthing unit or NICU (if applicable). Possible options that can be selected for this element are breastfeeding, skin-to-skin care, sucrose, other, no pain relief measures, no heel prick sampling, and unknown if pain relief was provided. The element is multi-select so multiple pain relief methods can be selected in combination; however, validation rules in the BIS prevent the last 3 options from being selected at the same time as one of the first 4 pain relief measures. At the stage of analysis and interpretation, the study team decided to combine the options breastfeeding and skin-to-skin care together into one category representing ‘parent-led’ approaches to newborn pain treatment, since both of these methods require parental involvement for implementation.

Descriptive statistics and chi square tests were used to compare pre- and post-intervention changes in the proportion of infants who received any type(s) of newborn pain treatment in each maternal–newborn unit. First, we combined the data for all eight sites together to examine overall changes in use of pain treatment before and after the intervention, and we also examined whether there were differences in the before-and-after changes for each site separately. Secondly, we examined whether there were any variations in pain treatment use, comparing before and after the intervention by several descriptive criteria selected a priori: neonatal levels of care, hospital birth volume, maternal parity, and infant sex. Prior to the analysis, results from the clinical surveys [14] revealed that one of the participating sites may have had challenges implementing the video due to changes in leadership, delays in intervention rollout and technical issues with data submission. In response to this, we modified our analysis plan to include a post hoc sensitivity analysis to assess the impact of excluding this site from the overall results. All analyses were conducted using SAS Version 9.4 (Cary, NC). For the overall study, ethical approval was obtained from the researchers’ host institution through Clinical Trials Ontario (Project#0832), and at each participating site. In accordance with the Personal Health Information Protection Act in Ontario, Canada, it was not possible or required to obtain consent from participants since we used only registry and administrative data in this analysis.

## 3. Results

### 3.1. Descriptive Characteristics of Participating Sites and Eligible Infants

**Figure 2:**
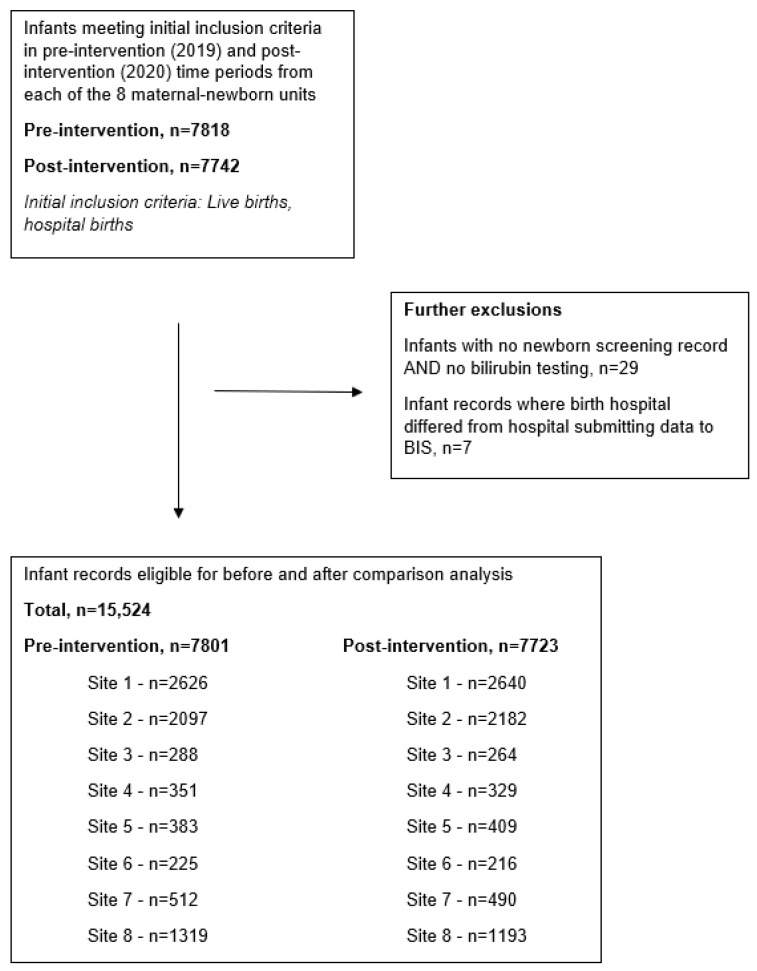
Study flow—data extracted from BORN Information System (BIS) on 1 April 2021.

Across the eight maternal–newborn units that participated in this study, there were 15,524 infants born during the pre- and post-intervention time periods who met the study criteria (*n* = 7801 and *n* = 7723, respectively) (Figure 2).

There was considerable variation in the number of infants born at each hospital, with sites ranging in size from 441 infants (2.8% of the study population) to 5266 infants (33.9% of the study population) (Table 1). The three largest sites (77.7% of the study population) had annual birth volumes of ≥2500, whereas the remaining five sites all had birth volumes of ≤1000 per year. For the level of care, four of the five smaller sites (*n* = 2675; 17.3% of the study population) were classified as newborn Level 1 (lowest-risk births), whereas the rest were classified as either Level 2b (two sites; *n* = 6791; 43.8% of the study population) or Level 2c (two sites; *n* = 6058; 39.0% of the study population). Nearly half (42.7%) of the infants in the study population had nulliparous mothers, and this was consistent across the eight maternal–newborn units. Similarly, there was little variation across the eight units in terms of infant sex, with just under half (48.2%) of the infants in the study population being female.

### 3.2. Before-and-After Results—Change in Use of Newborn Pain Treatment

**Figure 3:**
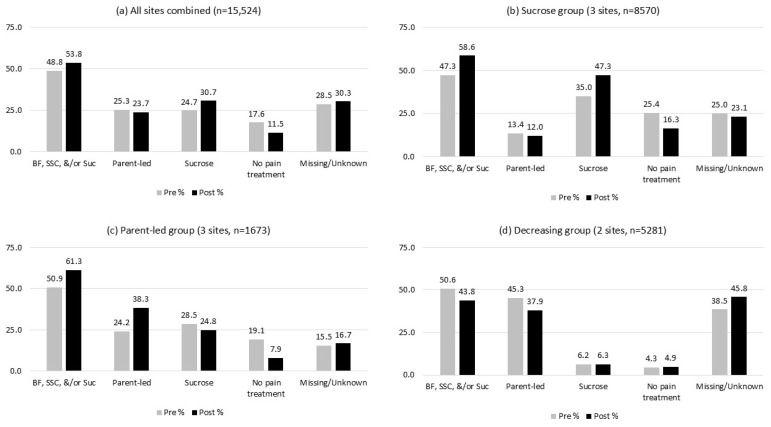
Change in proportion of infants who received pain treatment pre- and post-implementation of the intervention, all sites combined (**a**) and by specific clusters of sites (**b**–**d**). BF = breastfeeding, SSC = skin-to-skin care, Suc = sucrose, Parent-led = breastfeeding and/or skin-to-skin care.

In Table 2, we present the before-and-after results for all the possible options in the BIS pain management data element; however, our primary focus was on changes to the proportion of infants who received any of the three evidence-based pain treatment methods. Combining the results from all eight maternal–newborn units in the study population, there was a positive change of 5% in the proportion of newborns receiving any pain treatment (breastfeeding, skin-to-skin care, and/or sucrose), increasing from 48.8% of the infants pre-intervention to 53.8% of the infants post-intervention (*p* < 0.0001) (Table 2, Figure 3a). In parallel, there was a corresponding decrease of 6.1% in the proportion of newborns receiving no pain treatment pre- and post-intervention (17.6% to 11.5%, *p* < 0.0001). This overall increase in pain treatment use appeared to be largely driven by changes in sucrose use, which increased from 24.7% of the infants pre-intervention to 30.7% of the infants post-intervention (*p* < 0.0001). Of note, there was also a high amount of missing data for the pain management outcome (option selected = ‘unknown if pain relief was provided’), which increased slightly from 28.5% pre-intervention to 30.3% post-intervention.

Next, we examined whether there was variation in the before-and-after changes across each maternal–newborn unit separately, and by the hospital birth volume, neonatal level of care, parity, and infant sex. As can be seen in Table 2, the change in pain treatment use was not consistent across the eight sites, with significant differences observed between the maternal–newborn units for all three types of pain treatment as well as the use of no pain treatment (*p* < 0.0001). To summarize the variation, five sites (1, 3, 4, 6, 8) had increases in the use of any of the three types of pain treatment, four sites (1, 3, 4, 6) had increases in use of parent-led pain treatment (breastfeeding and/or skin-to-skin care), and three sites (1, 5, 8) had increases in use of sucrose. Further highlighting the variation between the maternal–newborn units, two sites (2, 7) had decreases in the use of any pain treatment after the intervention and four sites (2, 5, 7, 8) had considerable decreases in the use of parent-led pain treatment specifically.

There were also some important differences in pain treatment use before and after the intervention by hospital birth volume and neonatal level of care. In comparison with the smaller units, the maternal–newborn units with annual birth volumes ≥2500 had a large increase in sucrose use (7.5%) after the intervention (23.2% to 30.7%), and thus had increased use of any pain treatment overall (43.5% to 49.1%). Similarly, the sucrose use went up by 12.5% at the Level 2c sites (34.8% to 47.3%), whereas the Level 1 sites had increased use of parent-led pain treatment (7.3%, from 45.2% to 52.5%). The Level 1 and Level 2c sites also had substantial decreases in the proportion of infants receiving no pain treatment, whereas the Level 2b sites had little change in no pain treatment use and a large decrease in the use of parent-led approaches (30.2% to 23.5%). Notably, there were no statistically significant differences in the use of newborn pain treatment before and after the intervention in relation to the parity or infant sex, which suggests that the variation in pain treatment outcomes is primarily due to differences between the sites themselves rather than maternal or infant characteristics (Table 2).

### 3.3. Interpreting Patterns of Change in Newborn Pain Management

To facilitate the interpretation and understanding of the different patterns of change observed across the eight maternal–newborn units in our study, we decided to organize the sites into three distinct groups based on the similarity of their results before and after the intervention (Figure 3). The first grouping (*n* = 8570; Figure 3b) can be interpreted as the “sucrose” category (sites 1, 5, 8). The maternal–newborn units in this group tended to have larger birth volumes and higher levels of care (2b, 2c) and their increased use of sucrose for newborn pain treatment was often used instead of parent-led approaches (sites 5 and 8 in particular). The second grouping, which we interpret as the “parent-led” category (*n* = 1673; Figure 3c), was made up of the three smallest maternal–newborn units (sites 3, 4, 6). All of these sites were classified as Level 1 (lowest-risk births) and had considerable increases in parent-led pain treatment post-intervention (24.2% before to 38.3% after). The third grouping (sites 2, 7) can be interpreted as the “decreasing” category (*n* = 5281; Figure 3d), as these maternal–newborn units saw their use of newborn pain treatment fall after the implementation period. This grouping comprised two diverse maternal–newborn units with different levels of care (Level 1 (site 7) and 2b (site 2)) as well as very different birth volumes (*n* = 1002 and *n* = 4279, respectively). Both sites in the decreasing category, but especially site 2, also had increases in the amount of ‘unknown’ data for newborn pain treatment over the course of the study, which makes the interpretation of their results more challenging. This is important to note since site 2 was one of the largest sites included in the study and thus strongly influenced the overall combined before-and-after results.

To assess the magnitude of the impact of this one hospital on the overall results, we conducted a post hoc sensitivity analysis excluding site 2 from the study. Under this scenario, we re-ran the before-and-after comparison on the remaining seven sites and found that the overall increase in the use of any newborn pain treatment was 9.6%, from 50.9% of the infants pre-intervention to 60.5% of the infants post-intervention (*p* < 0.0001), which is nearly double the overall increase of 5.0% that was actually observed when site 2 was included.

## 4. Discussion

The findings of this study highlighted a small but statistically significant increase in the use of any newborn pain treatment (breastfeeding, skin-to-skin care, and/or sucrose) during newborn blood sampling 6 months after the implementation of a parent-targeted and parent-mediated video intervention in maternal–newborn care settings. However, the changes varied between the participating maternal–newborn units. For example, in the three smallest units with lower levels of acuity, the use of the parent-led strategies of breastfeeding or skin-to-skin care increased from baseline, and in three of the larger units with higher levels of acuity, the use of sucrose increased from baseline. These increases in the use of pain management during painful procedures in six of the eight units are promising and align with recommendations in the new Canadian national pediatric pain standard to use breastfeeding, kangaroo care, and sucrose for infants for acute procedural pain (recently published in 2023) [7].

Our results are similar to those of other studies where sucrose is more frequently used than parent-led strategies in units where sick newborns are cared for [21]. For example, another Canadian study reported poor uptake of parent-led interventions during painful procedures in NICU settings [1] and similarly, Australian parents of babies who had been in a NICU also reported minimal involvement with their newborns during painful procedures [22]. In these cases, parent-led pain relief strategies may not be as readily available because the parent or parents may not be present at the time of treatment. Similarly, our findings suggest that using sucrose for newborn pain treatment may be easier and preferred in sites with larger birth volumes and more complex births (indicated by higher levels of care), whereas breastfeeding and skin-to-skin care could be more feasible to implement in smaller, more low-risk settings, where the parent or parents are more likely to be present. Numerous published reports focus on clinicians’ barriers to involving parents in newborn pain management, highlighting the ongoing challenges in making substantial improvement in newborn pain care [23,24,25,26,27]. In addition, another possible explanation for the increase in sucrose use may be due to the Hawthrone effect [28], whereby nurses recognize that they are being studied and that certain practices (e.g., sucrose administration) are expected of them.

Despite improvements in pain management in six of the participating sites, the other two participating sites showed decreases in newborn pain treatment after the intervention. One of the two ‘decreasing’ sites already had a high proportion (80.7%) of infants receiving parent-led pain treatment *prior* to the video intervention (Table 2), so had perhaps already reached the ‘ceiling’ of newborn pain treatment in their unit. The results from the clinical survey portion of this study [14] support this as staff from this site reported that parent-led pain relief strategies were already being used in their unit and most nurses were already verbally explaining these strategies to parents before the video intervention. The second site in this group (site 2) reported other challenges such as changes in leadership and delays in the rollout of the intervention in their clinical staff survey. They also faced several unexpected technical issues involving overall data entry into the BIS for several months in 2020 and 2021 and experienced a large increase in ‘unknown’ data for newborn pain treatment over the course of the study.

Another possible reason for the variability in the results may be that in some units, heel pricks are performed by laboratory staff or phlebotomists [13,14]. Although there is little published information about phlebotomists’ role in newborn pain management, a study of phlebotomists’ knowledge and use of comforting measures in pediatric care highlighted that phlebotomists receive minimal education in pediatric pain management and may be less supportive of parent-led approaches to newborn pain relief [29]. In a study of interprofessional collaboration regarding procedural pain in newborns, parents were perceived by clinicians as playing a limited role in their newborn’s pain management, despite parents wishing to be more involved [26]. In our other studies, we also reported nurses’ perspectives that when phlebotomists performed the heel lances, parents’ involvement during the procedure was limited [14]. This highlights the need for all clinicians involved in neonatal care to be engaged and included in unit- and organization-wide educational interventions.

Although our results show overall improvements in pain care following the introduction of the parent-targeted video, attributing these changes exclusively to the video needs to be interpreted with caution. In recent years, there has been an increasing focus on pain in children, including newborns, especially in Canada, as illustrated by the publication of the Canadian national pediatric pain standard in 2023 [7]. Such initiatives occurring within the same time period as this study may have contributed to the observed improvements in the use of pain management strategies [30]. In addition, although the introduction of the video was the target intervention, multifaceted intervention strategies were used to promote the video and the study; therefore, we do not know if the changes were attributed to other aspects, such as engagement with the study, for example, through the community of practice sessions.

### Strengths and Limitations

The key strength of this study is that this analysis makes use of systematically collected data from a province-wide birth registry, which means we have a complete sample (census) of infants in each participating maternal–newborn unit rather than a random or convenience sample. The eight participating sites were also diverse in terms of the birth volumes, urban and rural locations, and different levels of care across the province.

There were, however, several limitations. Firstly, the recruitment of sites to the larger study was challenging at the outset, with the participation of only 8 of the eligible 47 units [13]. In addition, the post-implementation time period in 2020 largely coincided with the beginning of the COVID-19 pandemic. This resulted in the temporary cessation of research, and many sudden changes and additional pressures during the birth experience for both healthcare staff and families as new procedures and care routines were quickly put in place. In Ontario, this included practice changes around breastfeeding and other newborn care as well as the restriction of hospital visitors and alterations to cesarean delivery protocols [31]. These disruptions may have influenced the ability of maternal–newborn units to implement practice change in general, but also parent-led pain treatment in particular, which may also explain why most of the increases we observed were due to sucrose use. Other limitations include the relatively high amount of missing data reported for newborn pain treatment, especially in one of the participating sites. Despite the newborn pain data element being functional in the BIS since 2014, a lack of systematic documentation of pain management strategies remains challenging when trying to collect such data, like in other settings [32,33,34]. We also did not analyze data beyond 6 months after the intervention, so we are unable to conclude whether the increase in the use of pain management strategies was sustained over the long term. Similarly, we did not examine whether there were changes in newborn pain treatment over the same time period in other hospitals across the province that did not participate in our study. Fortunately, it could be possible to examine this in the future due to the ongoing systematic data collection in the BIS. Future research is warranted to explore the effectiveness of different intervention strategies to support video use with different populations and contexts of care.

## 5. Conclusions

This pre–post study highlighted that following the implementation of the brief parent-targeted video demonstrating breastfeeding, skin-to-skin care, and sucrose use during newborn blood sampling, there was an increase in the overall use of these pain treatment strategies. However, the overall increase was small, and there was variability across the eight units. Further research is warranted to explore effective and sustained ways to improve newborn pain management, including parents’ involvement during painful procedures.

## Figures and Tables

**Table 1:** Descriptive characteristics for participating sites and infants born at each site during pre- and post-intervention periods.

	Maternal–Newborn Unit																Total	
	Site 1		Site 2		Site 3		Site 4		Site 5		Site 6		Site 7		Site 8			
	*n*	% (Row)	*n*	% (Row)	*n*	% (Row)	*n*	% (Row)	*n*	% (Row)	*n*	% (Row)	*n*	% (Row)	*n*	% (Row)	*n*	% (Row)
Total infants in study	5266	33.9	4279	27.6	552	3.6	680	4.4	792	5.1	441	2.8	1002	6.5	2512	16.2	15,524	100.0
Hospital birth volume (annual)																		
≤1000	0	0.0	0	0.0	552	15.9	680	19.6	792	22.8	441	12.7	1002	28.9	0	0.0	3467	100.0
≥2500	5266	43.7	4279	35.5	0	0.0	0	0.0	0	0.0	0	0.0	0	0.0	2512	20.8	12,057	100.0
Newborn level of care (LOC)																		
Level 1	0	0.0	0	0.0	552	20.6	680	25.4	0	0.0	441	16.5	1002	37.5	0	0.0	2675	100.0
Level 2b	0	0.0	4279	63.0	0	0.0	0	0.0	0	0.0	0	0.0	0	0.0	2512	37.0	6791	100.0
Level 2c	5266	86.9	0	0.0	0	0.0	0	0.0	792	13.1	0	0.0	0	0.0	0	0.0	6058	100.0
	*n*	% (Col)	*n*	% (Col)	*n*	% (Col)	*n*	% (Col)	*n*	% (Col)	*n*	% (Col)	*n*	% (Col)	*n*	% (Col)	*n*	% (Col)
Parity																		
0	2305	43.8	1812	42.3	239	43.3	260	38.2	324	40.9	160	36.3	404	40.3	1132	45.1	6636	42.7
1	1965	37.3	1434	33.5	224	40.6	218	32.1	270	34.1	166	37.6	355	35.4	815	32.4	5447	35.1
2+	996	18.9	1030	24.1	89	16.1	202	29.7	194	24.5	115	26.1	243	24.3	565	22.5	3434	22.1
Missing	0	0.0	3	0.1	0	0.0	0	0.0	4	0.5	0	0.0	0	0.0	0	0.0	7	0.0
Infant sex																		
Female	2487	47.2	2045	47.8	267	48.4	321	47.2	391	49.4	217	49.2	519	51.8	1234	49.1	7481	48.2
Male	2779	52.8	2234	52.2	285	51.6	359	52.8	401	50.6	224	50.8	483	48.2	1278	50.9	8043	51.8

**Table 2:** Change in type(s) of newborn pain treatment received by infants before (pre-) and after (post-) implementation of the intervention.

		Type(s) of Newborn Pain Treatment Received by Infants															
		Any of 3 Types		Parent-Led		Sucrose		Other		No Pain Relief		No Heel Prick		Unknown if Pain Relief Provided		Total	
		*n*	% (Row)	*n*	% (Row)	*n*	% (Row)	*n*	% (Row)	*n*	% (Row)	*n*	% (Row)	*n*	% (Row)	*n*	% (Row)
Overall (all sites combined)	Pre	3806	48.8	1971	25.3	1923	24.7	283	3.6	1376	17.6	136	1.7	2222	28.5	7801	100.0
	Post	4152	53.8	1829	23.7	2373	30.7	221	2.9	889	11.5	138	1.8	2339	30.3	7723	100.0
*p*-value		<0.0001		0.1353		<0.0001				<0.0001							
By maternal–newborn unit (MNU)																	
Site 1	Pre	1000	38.1	224	8.5	782	29.8	31	1.2	904	34.4	18	0.7	678	25.8	2626	100.0
	Post	1438	54.5	344	13.0	1115	42.2	20	0.8	529	20.0	3	0.1	656	24.8	2640	100.0
Site 2	Pre	901	43.0	769	36.7	156	7.4	150	7.2	110	5.2	9	0.4	930	44.3	2097	100.0
	Post	798	36.6	640	29.3	167	7.7	110	5.0	128	5.9	20	0.9	1133	51.9	2182	100.0
Site 3	Pre	123	42.7	109	37.8	23	8.0	36	12.5	31	10.8	16	5.6	86	29.9	288	100.0
	Post	141	53.4	139	52.7	3	1.1	25	9.5	14	5.3	28	10.6	56	21.2	264	100.0
Site 4	Pre	256	72.9	40	11.4	222	63.2	5	1.4	37	10.5	28	8.0	27	7.7	351	100.0
	Post	260	79.0	78	23.7	195	59.3	1	0.3	13	4.0	30	9.1	26	7.9	329	100.0
Site 5	Pre	321	83.8	95	24.8	266	69.5	6	1.6	5	1.3	5	1.3	50	13.1	383	100.0
	Post	332	81.2	9	2.2	326	79.7	2	0.5	30	7.3	18	4.4	27	6.6	409	100.0
Site 6	Pre	61	27.1	60	26.7	1	0.4	12	5.3	97	43.1	35	15.6	21	9.3	225	100.0
	Post	95	44.0	93	43.1	3	1.4	8	3.7	37	17.1	23	10.6	53	24.5	216	100.0
Site 7	Pre	418	81.6	413	80.7	7	1.4	6	1.2	1	0.2	15	2.9	74	14.5	512	100.0
	Post	372	75.9	372	75.9	0	0.0	9	1.8	4	0.8	15	3.1	91	18.6	490	100.0
Site 8	Pre	726	55.0	261	19.8	466	35.3	37	2.8	191	14.5	10	0.8	356	27.0	1319	100.0
	Post	716	60.0	154	12.9	564	47.3	46	3.9	134	11.2	1	0.1	297	24.9	1193	100.0
*p*-value		<0.0001		<0.0001		<0.0001				<0.0001							
By hospital birth volume																	
≤1000	Pre	1179	67.0	717	40.8	519	29.5	65	3.7	171	9.7	99	5.6	258	14.7	1759	100.0
	Post	1200	70.3	691	40.5	527	30.9	45	2.6	98	5.7	114	6.7	253	14.8	1708	100.0
≥2500	Pre	2627	43.5	1254	20.8	1404	23.2	218	3.6	1205	19.9	37	0.6	1964	32.5	6042	100.0
	Post	2952	49.1	1138	18.9	1846	30.7	176	2.9	791	13.2	24	0.4	2086	34.7	6015	100.0
*p*-value		0.0433		0.3710		0.0003				0.3133							
By newborn level of care (LOC)																	
Level 1	Pre	858	62.4	622	45.2	253	18.4	59	4.3	166	12.1	94	6.8	208	15.1	1376	100.0
	Post	868	66.8	682	52.5	201	15.5	43	3.3	68	5.2	96	7.4	226	17.4	1299	100.0
Level 2b	Pre	1627	47.6	1030	30.2	622	18.2	187	5.5	301	8.8	19	0.6	1286	37.6	3416	100.0
	Post	1514	44.9	794	23.5	731	21.7	156	4.6	262	7.8	21	0.6	1430	42.4	3375	100.0
Level 2c	Pre	1321	43.9	319	10.6	1048	34.8	37	1.2	909	30.2	23	0.8	728	24.2	3009	100.0
	Post	1770	58.1	353	11.6	1441	47.3	22	0.7	559	18.3	21	0.7	683	22.4	3049	100.0
*p*-value		<0.0001		<0.0001		<0.0001				<0.0001							
By parity																	
0	Pre	1588	48.3	788	24.0	840	25.6	108	3.3	570	17.4	39	1.2	989	30.1	3285	100.0
	Post	1773	52.9	728	21.7	1065	31.8	85	2.5	385	11.5	30	0.9	1086	32.4	3351	100.0
1	Pre	1381	50.1	738	26.7	676	24.5	92	3.3	523	19.0	53	1.9	717	26.0	2759	100.0
	Post	1476	54.9	683	25.4	810	30.1	85	3.2	337	12.5	60	2.2	737	27.4	2688	100.0
2+	Pre	835	47.6	445	25.4	405	23.1	83	4.7	283	16.1	44	2.5	515	29.4	1754	100.0
	Post	900	53.6	418	24.9	495	29.5	51	3.0	166	9.9	48	2.9	516	30.7	1680	100.0
Missing	Pre	2	66.7	0	0.0	2	66.7	0	0.0	0	0.0	0	0.0	1	33.3	3	100.0
	Post	3	75.0	0	0.0	3	75.0	0	0.0	1	25.0	0	0.0	0	0.0	4	100.0
*p*-value		0.6661		0.9793		0.7105				0.4885							
By infant sex																	
Female	Pre	1860	48.9	991	26.1	905	23.8	132	3.5	684	18.0	76	2.0	1056	27.8	3800	100.0
	Post	1990	54.1	871	23.7	1146	31.1	106	2.9	414	11.2	65	1.8	1112	30.2	3681	100.0
Male	Pre	1946	48.6	980	24.5	1018	25.4	151	3.8	692	17.3	60	1.5	1166	29.1	4001	100.0
	Post	2162	53.5	958	23.7	1227	30.4	115	2.8	475	11.8	73	1.8	1227	30.4	4042	100.0
*p*-value		0.4012		0.1016		0.4217				0.1442							

## Data Availability

We are not able to share data with the public due to the Personal Health Information Protection Act in Ontario, Canada. The data analyzed during this study are held securely at the prescribed registry BORN Ontario. Data sharing regulations prevent these data from being made available publicly due to the personal health information in the datasets. Enquiries regarding BORN data must be directed to BORN Ontario (Science@BORNOntario.ca).
